# Scalp Biopsies for Alopecia

**DOI:** 10.3390/dermatopathology13030044

**Published:** 2026-09-16

**Authors:** Tullio Brunetti, Cosimo Misciali, Luca Rapparini, Francesca Bruni, Michela Starace, Michelangelo La Placa

**Affiliations:** 1 Dermatology Unit, IRCCS Azienda Ospedaliero-Universitaria di Bologna, Via Massarenti 1, 40138 Bologna, Italy; tullio.brunetti@studio.unibo.it (T.B.); cosimo.misciali@aosp.bo.it (C.M.); luca.rapparini5@unibo.it (L.R.); francesca.bruni3@studio.unibo.it (F.B.); michela.starace2@unibo.it (M.S.); 2 Department of Medicine and Surgery (DIMEC), Alma Mater Studiorum University of Bologna, Via Massarenti 9, 40138 Bologna, Italy

**Keywords:** alopecia, biopsy, scalp, histopathology, trichoscopy, scarring alopecia, cicatricial alopecia, review

## Abstract

Background: Hair loss is one of the most common reasons patients seek dermatologic evaluation. Although clinical examination and trichoscopy establish the diagnosis in most cases, scalp biopsy remains essential when findings are inconclusive, when cicatricial alopecia is suspected, or when histopathologic documentation is required before systemic therapy. Methods: This narrative review synthesizes evidence from PubMed/MEDLINE and Embase on the indications, biopsy site selection, sampling technique, specimen processing, and histopathologic interpretation of scalp biopsy in alopecia. Results: Diagnostic yield depends on appropriate patient selection, trichoscopy-guided site selection, meticulous sampling, and optimal processing. A 4-mm punch oriented parallel to the hair shafts and extending into the subcutaneous fat, combined with horizontal and vertical sectioning (HoVert or Tyler technique), allows the most comprehensive assessment of follicular architecture. Histopathology reliably separates scarring from nonscarring alopecia and, in cicatricial forms, classifies disease by the predominant inflammatory infiltrate following the NAHRS scheme. Ancillary studies, including direct immunofluorescence, periodic acid-Schiff staining, elastic fiber stains, and microbiologic cultures, are applied selectively when clinically indicated. Conclusions: Integrating clinical, trichoscopic, and histopathologic findings improves diagnostic accuracy and guides therapeutic decisions. Close collaboration between the dermatologist and dermatopathologist maximizes the diagnostic value of scalp biopsy.

## Simple Summary

Hair loss is a common condition with a wide range of underlying causes. In many cases, dermatologists can establish the diagnosis through clinical examination of the scalp, supplemented by dermoscopy, which enables detailed visualization of the hair shafts and scalp surface. However, the underlying cause may occasionally remain uncertain, or there may be concern for a scarring (cicatricial) alopecia. In such cases, scalp biopsy with histopathological examination is indicated. This article reviews the clinical indications for scalp biopsy, as well as the optimal biopsy technique and site selection. It also highlights the importance of close collaboration between the dermatologist and the dermatopathologist in achieving accurate diagnoses and guiding appropriate management of patients with scalp disorders.

## 1. Introduction

Scalp disorders are among the most common reasons for consultation in dermatologic practice. They comprise a heterogeneous group of conditions with distinct pathogenetic mechanisms, clinical presentations, and natural histories. Androgenetic alopecia (AGA), the most common form of hair loss, affects approximately 50% of men by the age of 50 and 40% of women by the age of 70; although hair loss is frequently perceived primarily as a cosmetic concern, it may affect quality of life in a proportion of patients [1,2,3]. Although clinical examination and trichoscopy are sufficient to establish the diagnosis in many cases, histopathologic evaluation remains indispensable when findings are inconclusive, when a scarring process is suspected, or when objective histopathologic confirmation is required before initiating long-term systemic therapy [3,4,5]. The diagnostic value of a scalp biopsy depends largely on the integration of clinical, trichoscopic, and histopathologic findings. This multidisciplinary approach is particularly important in primary cicatricial alopecia, a heterogeneous group of inflammatory disorders characterized by irreversible destruction of hair follicles and their replacement by fibrous tissue [6,7]. Early diagnosis is essential because timely recognition of active disease may prevent further irreversible hair loss. Providing the dermatopathologist with detailed clinical information is therefore crucial. Diagnostic accuracy is significantly improved when biopsy specimens are accompanied by a comprehensive clinical history, trichoscopic findings, and photographs documenting the biopsy site [4]. Primary cicatricial alopecias include lichen planopilaris (LPP), frontal fibrosing alopecia (FFA), discoid lupus erythematosus (DLE), folliculitis decalvans (FD), central centrifugal cicatricial alopecia (CCCA), and dissecting cellulitis (DC) of the scalp. According to the North American Hair Research Society (NAHRS) classification, these disorders are categorized as lymphocytic, neutrophilic, mixed, or nonspecific based on the predominant inflammatory infiltrate [8,9,10,11,12,13]. Nevertheless, scarring alopecia may also occur secondary to other cutaneous disorders, including scalp sarcoidosis and, rarely, severe psoriatic involvement of the scalp [14,15]. By contrast, scalp involvement in dermatomyositis generally shows a nonscarring histopathologic pattern [16]. Because prognosis and therapeutic management differ substantially among these subtypes, accurate histopathologic classification has direct clinical implications [17,18,19,20]. In contrast, nonscarring alopecia, including AGA, alopecia areata (AA), telogen effluvium (TE), and trichotillomania, are usually diagnosed on the basis of clinical examination and trichoscopy, although scalp biopsy may be valuable in selected or diagnostically challenging cases [21,22,23]. Histopathologic examination is particularly useful when diffuse AA, TE, and female pattern hair loss (FPHL) present with overlapping clinical and trichoscopic features and cannot be reliably distinguished by noninvasive assessment alone [24,25]. The diagnostic yield of scalp biopsy depends on appropriate patient selection, accurate biopsy site selection, meticulous sampling technique, and optimal specimen processing. Biopsies obtained from inactive lesions or performed using suboptimal technique may provide limited or misleading information [4,25]. Conversely, a properly performed biopsy substantially improves diagnostic accuracy beyond that achieved by clinical evaluation alone and changes the working diagnosis often enough to influence both prognosis and therapeutic decision-making [4,26]. Two major technical advances have significantly enhanced the diagnostic performance of scalp biopsy. Horizontal sectioning, introduced in 1984, allows simultaneous evaluation of multiple hair follicles within a single section and quantitative assessment of follicular density, morphology, and hair-cycle distribution [22,27,28]. A later systematic review and meta-analysis found no significant difference in overall diagnostic rates between horizontal and vertical sectioning, although the available studies were heterogeneous and generally methodologically limited [29]. A subsequent advance was the introduction of trichoscopy-guided biopsy site selection, which addressed the equally important challenge of identifying the most informative area for sampling. In inflammatory alopecia, trichoscopic features such as perifollicular erythema, perifollicular scaling, broken hairs, black dots, and follicular tufting identify sites of active disease and therefore represent the optimal targets for biopsy [30,31,32,33].

## 2. Materials and Methods

This is a narrative review of current evidence on the indications, technique, and diagnostic value of scalp biopsy in alopecia.

A comprehensive search of PubMed/MEDLINE and Embase was performed from inception to February 2026. Keywords and MeSH terms included scalp biopsy, alopecia, cicatricial alopecia, hair loss, horizontal sections, transverse sections, trichoscopy, hair follicle histopathology, direct immunofluorescence (DIF), and dermatopathology. These terms were used individually and in combination with Boolean operators (AND, OR). The reference lists of key articles were screened manually to identify additional relevant publications.

Articles were screened by title and abstract, and those considered potentially relevant underwent full-text review. To be eligible, articles had to be published in peer-reviewed journals indexed in PubMed/MEDLINE or Embase, written in English, and focused on scalp biopsy or the histopathologic evaluation of alopecia. They also had to address at least one of the following topics: indications, site selection, technique, specimen processing, histopathologic patterns, ancillary diagnostic methods, or diagnostic pitfalls. Both original studies and reviews were eligible. Priority was given to seminal dermatopathology studies, systematic reviews, meta-analyses, and methodologically focused articles. Conference abstracts without a full-text version, non-English publications, and studies lacking adequate methodological detail were excluded.

The schematic illustration presented in Figure 7 was generated with the assistance of a generative artificial intelligence tool (ChatGPT, version 5.6; OpenAI, San Francisco, CA, USA). All authors reviewed and verified the accuracy of the illustration and take full responsibility for its content.

Study design, biopsy technique, histopathologic criteria, and diagnostic recommendations were synthesized narratively. Special emphasis was placed on biopsy site selection, technical considerations, and the interpretation of histopathological findings.

## 3. Histopathologic Patterns of Alopecia

The fundamental distinction on biopsy is between nonscarring and scarring alopecia. In scarring forms, destruction of follicular stem cells in the bulge region causes irreversible hair loss, so histopathologic classification is critical for management [6,34,35].

### 3.1. Nonscarring Alopecia

AGA is defined histopathologically by progressive follicular miniaturization and a shift toward vellus-like hairs. Horizontal sections outperform vertical ones both for diagnosis and for predicting therapeutic response [21,26,28]. Acute TE shows a preserved follicular count with a disproportionate increase in telogen hairs [21,36,37,38].

The histopathologic features of AA vary according to disease stage. Peribulbar lymphocytic inflammation (the “swarm of bees”) is characteristic of acute lesions but may be sparse or absent in later stages. Subacute lesions show increased catagen and telogen follicles, whereas chronic lesions are characterized predominantly by follicular miniaturization; nanogen follicles may provide an additional diagnostic clue [21,39,40,41,42].

**Figure 1:**
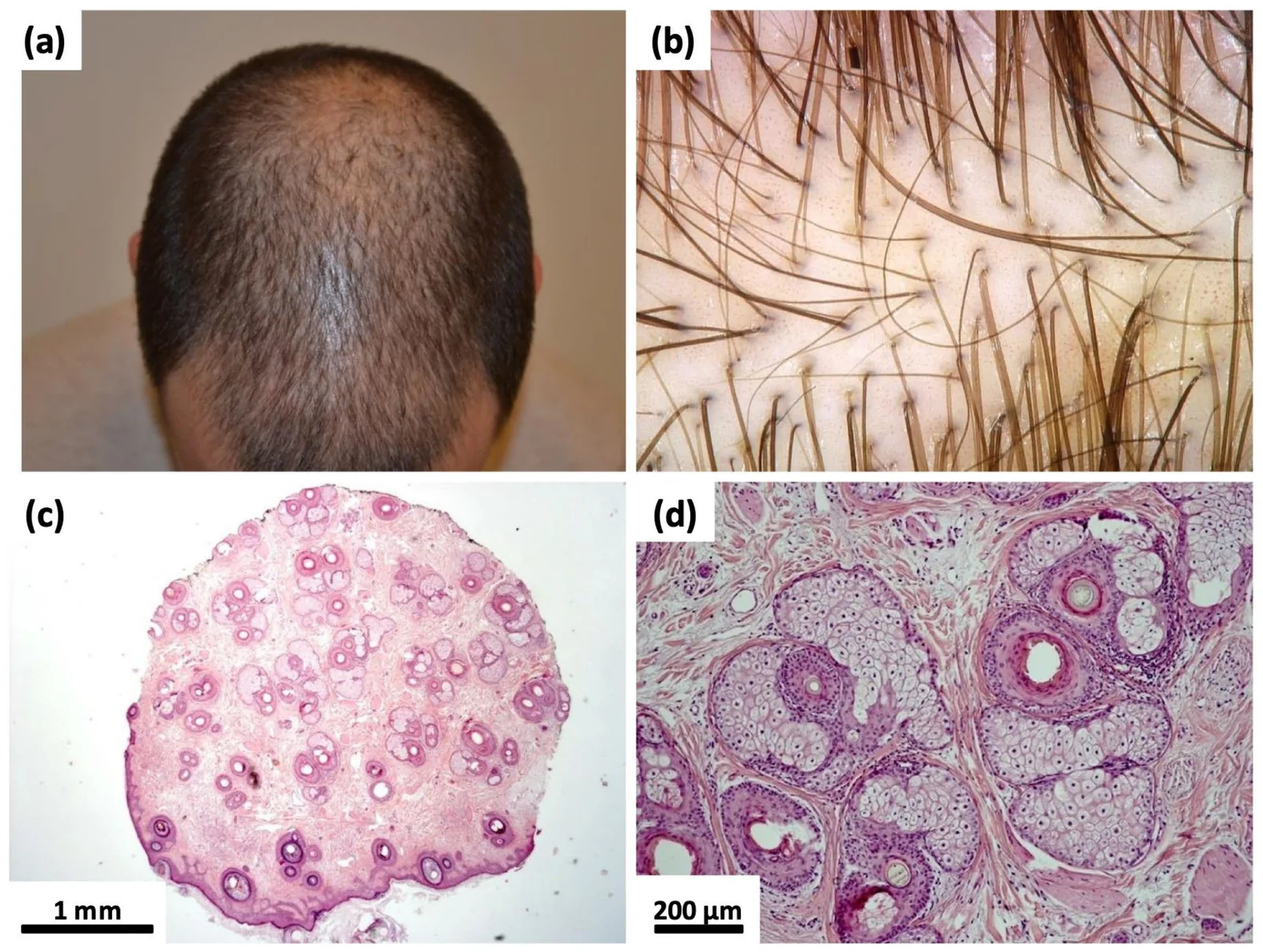
Androgenetic Alopecia. (**a**) Clinical examination: hair thinning involving the vertex and temporal regions. (**b**) Trichoscopy: hair-shaft diameter variability greater than 20%, reduced hair density per follicular unit and an increased proportion of vellus hairs. (**c**) Horizontal section of a 4-mm punch biopsy (4× magnification): follicular miniaturisation with numerous vellus-like follicles and a reduced terminal-to-vellus ratio. (**d**) Horizontal section (10× magnification): miniaturised follicles adjacent to preserved sebaceous lobules. Panels (**c**,**d**) haematoxylin and eosin. Scale bars: (**c**) 1 mm, (**d**) 200 µm. These images are from the personal histopathological collection of Dr. Cosimo Misciali (Dermatology Unit, IRCCS Azienda Ospedaliero-Universitaria di Bologna, Italy) and illustrate the scalp biopsy findings of nonscarring alopecia (androgenetic alopecia) described in the main text.

**Figure 2:**
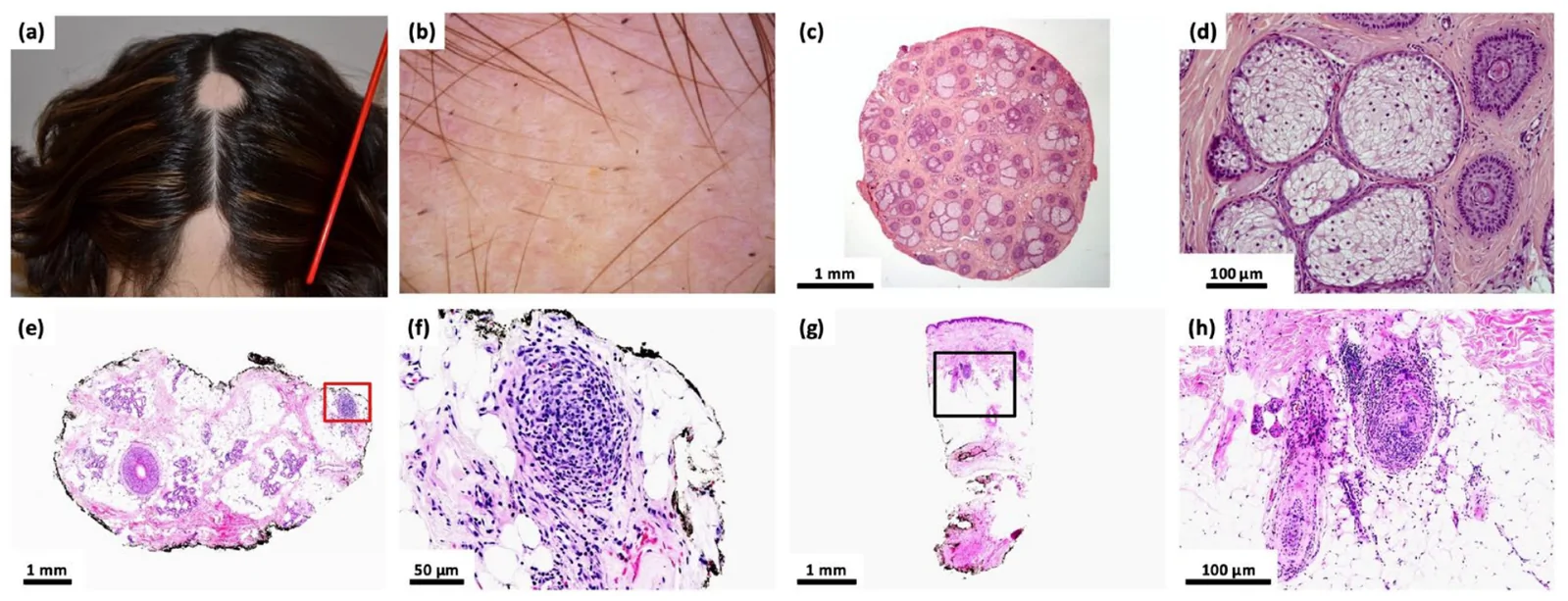
Alopecia areata. (**a**) Clinical presentation of patchy alopecia areata in a 32-year-old woman; the marker indicates the sampled patch. (**b**) Trichoscopy of the margin of the lesion: black dots, exclamation-mark and dystrophic hairs, together with yellow dots. (**c**) Horizontal section (4× magnification): preserved follicular units with an increased number of catagen and telogen follicles. (**d**) Miniaturised anagen (nanogen) follicles (20× magnification). (**e**) Horizontal section at the level of the subcutis (4× magnification); the red box marks the peribulbar region enlarged in (**f**). (**f**) “Swarm of bees”: mild-to-dense peribulbar lymphocytic infiltrate around the hair bulb (40× magnification). (**g**) Vertical section (4× magnification); the black box marks the region enlarged in (**h**). (**h**) Peribulbar lymphocytic infiltrate in the deep dermis (20× magnification). Panels (**c**–**h**) haematoxylin and eosin. Scale bars: (**c**) 1 mm, (**d**) 100 µm, (**e**) 1 mm, (**f**) 50 µm, (**g**) 1 mm, (**h**) 100 µm. These images are from the personal histopathological collection of Dr. Cosimo Misciali (Dermatology Unit, IRCCS Azienda Ospedaliero-Universitaria di Bologna, Italy) and illustrate the scalp biopsy findings of alopecia areata described in the main text.

**Figure 3:**
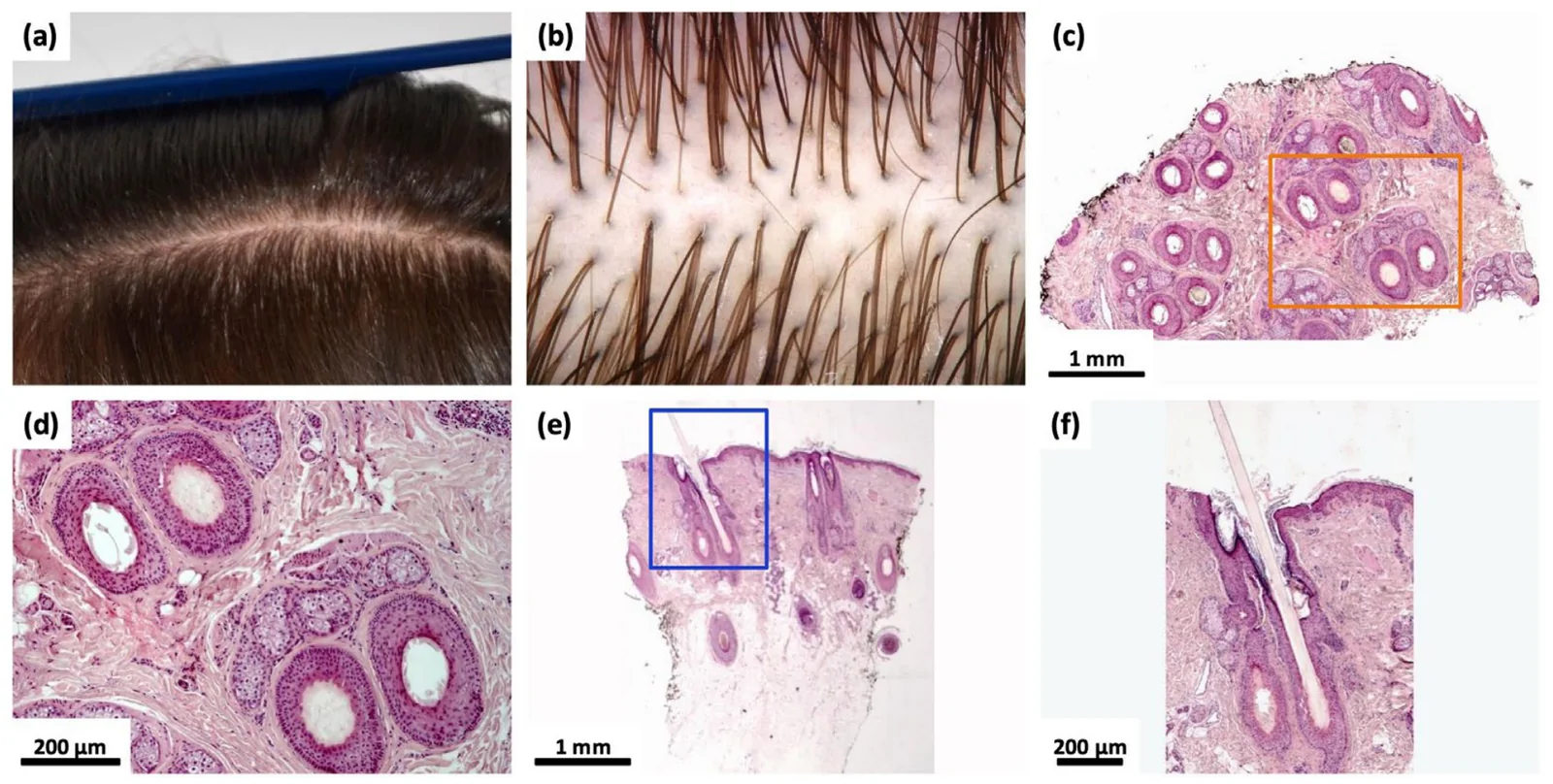
Acute telogen effluvium. (**a**) Clinical examination: mild diffuse thinning, most evident over the temporal regions. (**b**) Trichoscopy: absence of significant hair-shaft diameter variability, in contrast to androgenetic alopecia, with numerous short regrowing hairs distributed over both androgen-sensitive and androgen-independent areas. (**c**) Horizontal section (4× magnification); the orange box marks the follicular unit enlarged in (**d**). (**d**) Increased number of telogen follicles within a single follicular unit (10× magnification). (**e**) Vertical section (4× magnification); the blue box marks the region enlarged in (**f**). (**f**) Two telogen follicles in the superficial and mid dermis, the characteristic appearance of acute telogen effluvium (10× magnification). Panels (**c**–**f**) haematoxylin and eosin. Scale bars: (**c**) 1 mm, (**d**) 200 µm, (**e**) 1 mm, (**f**) 200 µm. These images are from the personal histopathological collection of Dr. Cosimo Misciali (Dermatology Unit, IRCCS Azienda Ospedaliero-Universitaria di Bologna, Italy) and illustrate the scalp biopsy findings of acute telogen effluvium described in the main text.

On horizontal sections, alopecia areata incognita (AAI), a diffuse variant of AA that can mimic TE, presents a more subtle picture: preserved follicular units, reduced numbers of terminal follicles, increased telogen structures with a mean count of 37%, a reduced terminal-to-vellus ratio averaging 3.3:1, telogen germinal units or small telogen follicles, and dilated infundibular openings [43,44]. Diffuse AA may show more intense peribulbar inflammation than patchy AA, including eosinophils and CD8+ T cells [44,45,46]. Scalp trichoscopy may reveal broken hairs and black dots, whereas examination of shed hair roots has demonstrated a sequential transition from anagen to catagen and TE, together with progressive proximal hair-shaft depigmentation correlated with disease duration (Figure 1, Figure 2 and Figure 3).

### 3.2. Traumatic Alopecia: Trichotillomania and Traction Alopecia

Traumatic alopecias include trichotillomania and traction alopecia (TA), which result from different forms of mechanical injury and show distinct clinicopathologic patterns. Trichotillomania, caused by repetitive self-induced pulling or manipulation of the hair, shows traumatic follicular changes, including distorted follicles, trichomalacia, pigment casts, empty follicular sheaths, and minimal inflammation [47,48]. TA, by contrast, results from chronic tensile forces and may progress to permanent follicular loss. In end-stage TA, horizontal sections may facilitate quantitative assessment of terminal and vellus follicles, typically showing loss of terminal follicles, fibrous tracts, and relative preservation of sebaceous glands [49,50]. The pauci-follicular unit (PFU), defined as a non-inflamed follicular unit with an intact sebaceous gland and fewer than two follicles, has been proposed as a useful histopathologic clue to TA [50]. The finding of three or more PFUs in a single 4-mm punch biopsy, evaluated on horizontal sections, was proposed as a practical shortcut sufficient to identify TA [50]. This criterion is particularly useful when TA coexists with other forms of hair loss, a relatively common situation in which counting PFUs helps to separate the traction component from the overall histopathologic picture.

Trichotillomania is an important differential diagnosis of AA, since both may present with patchy, nonscarring hair loss. The distinction rests on the type of follicular change rather than on its extent. AA shows a peribulbar lymphocytic infiltrate with an increased proportion of catagen and telogen follicles, whereas trichotillomania shows the changes associated with mechanical injury: distorted follicles, traumatized hair bulbs, trichomalacia, pigment casts, and empty follicular sheaths, with minimal or absent inflammation [43,47,48,51]. Because an increased number of catagen follicles occurs in both conditions, hair-shaft damage, trichomalacia, and pigment casts, rather than the hair-cycle shift alone, are the findings that point to a traumatic cause [47,48].

### 3.3. Scarring Alopecia

Across all primary cicatricial alopecia, the histopathologic hallmarks are the same: follicular destruction, replacement by fibrous tracts, loss of sebaceous glands, and eccrine duct dilation [6,7,10,52]. The NAHRS classification groups these disorders by the predominant inflammatory cell type [9,10,11].

**Figure 4:**
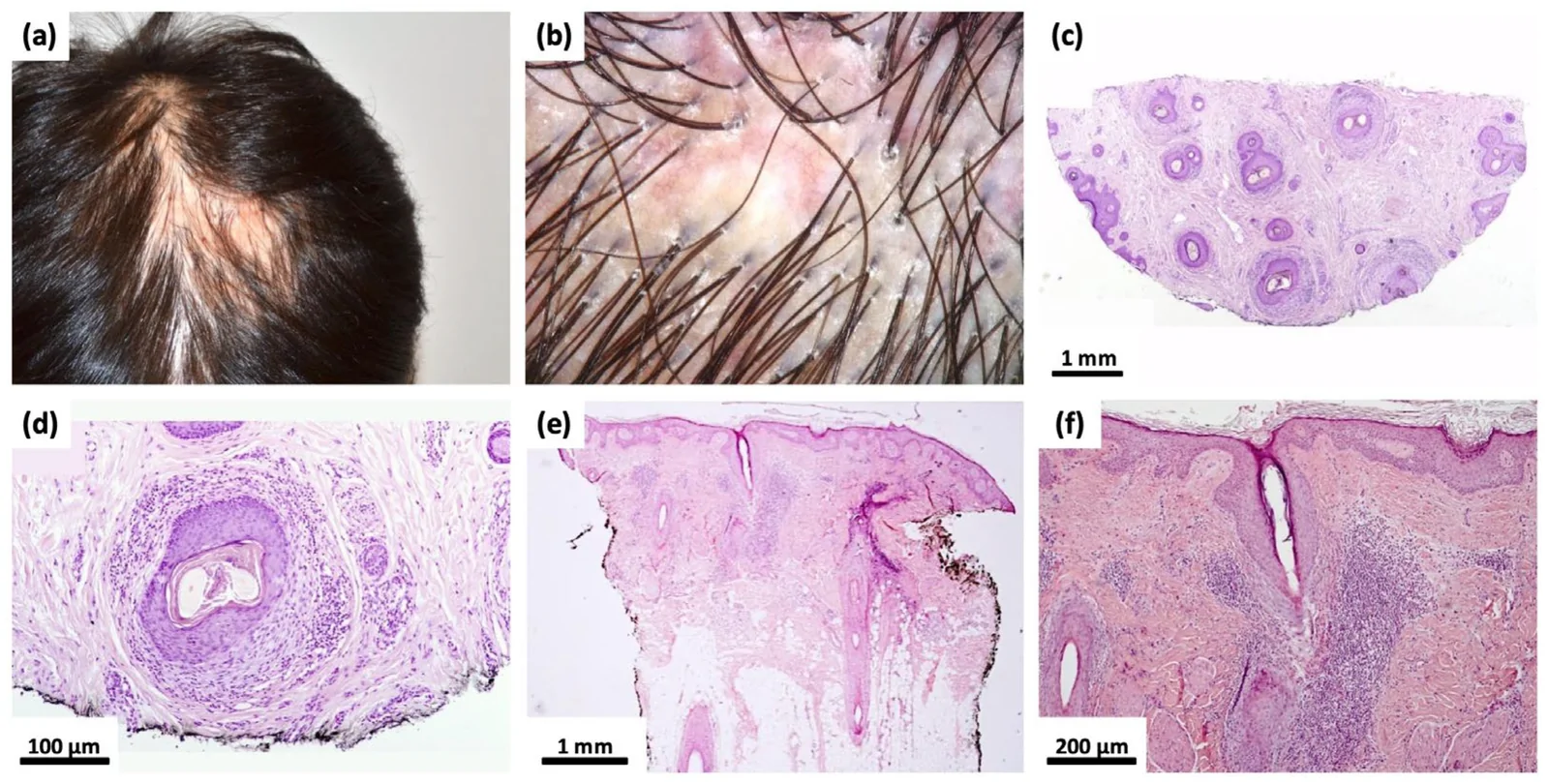
Lichen planopilaris. (**a**) Clinical image: patchy alopecia predominantly involving the vertex. (**b**) Trichoscopy: loss of follicular openings, perifollicular hyperkeratosis, and erythematous areas alternating with white structureless areas. (**c**) Vertical section (4× magnification): dense peri-isthmus lymphocytic infiltrate with perifollicular lamellar fibrosis and loss of sebaceous glands. (**d**) The same features at higher magnification (20× magnification). (**e**) Horizontal section (4× magnification): lichenoid interface change at the infundibulum with concentric perifollicular fibrosis. (**f**) Vertical section (10× magnification): lichenoid interface change at the infundibulum and isthmus. Panels (**c**–**f**) haematoxylin and eosin. Scale bars: (**c**) 1 mm, (**d**) 100 µm, (**e**) 1 mm, (**f**) 200 µm. These images are from the personal histopathological collection of Dr. Cosimo Misciali (Dermatology Unit, IRCCS Azienda Ospedaliero-Universitaria di Bologna, Italy) and illustrate the scalp biopsy findings of lichen planopilaris described in the main text.

**Figure 5:**
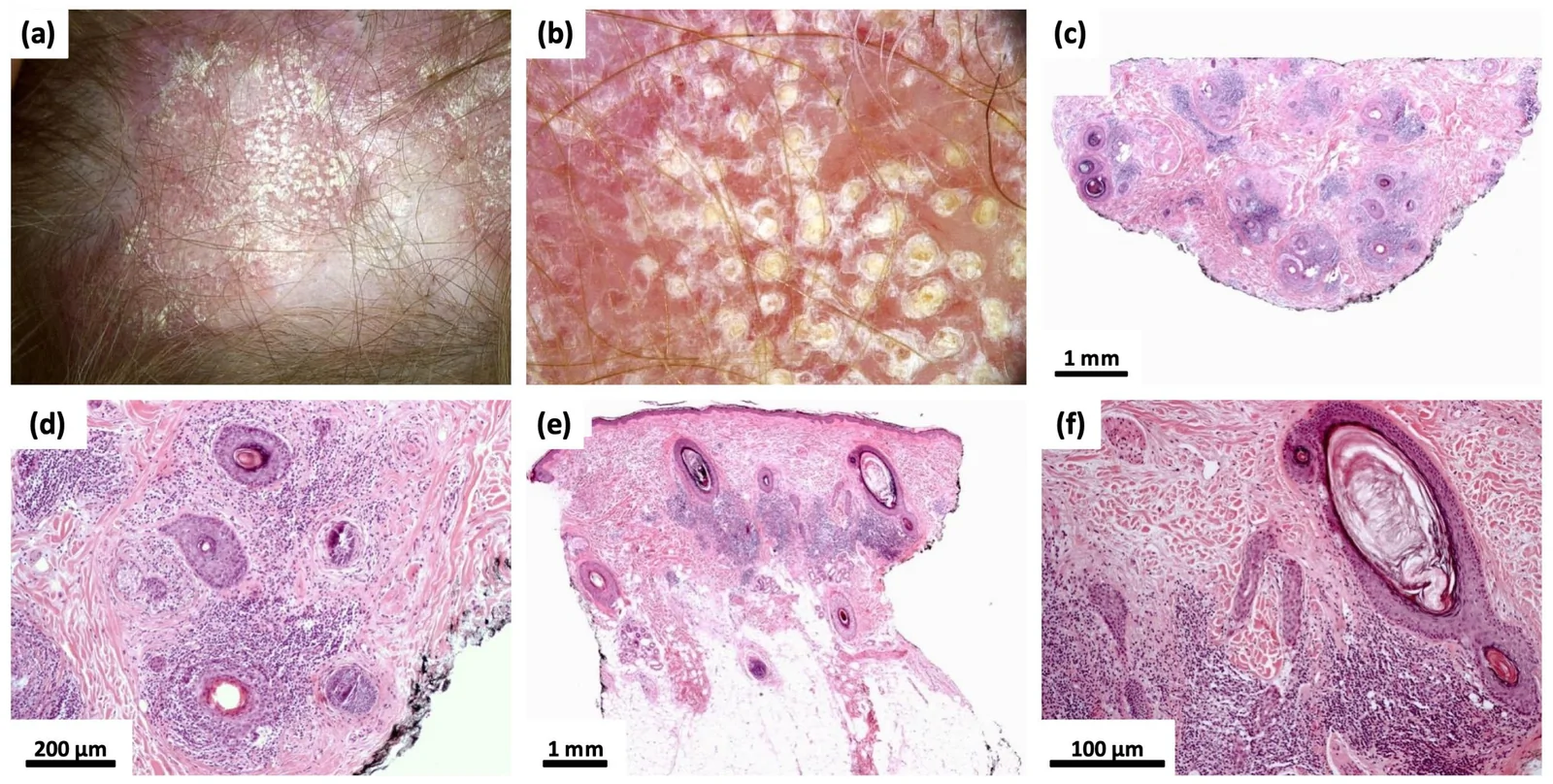
Discoid lupus erythematosus. (**a**) Clinical image: scarring alopecia of the scalp. (**b**) Trichoscopy: follicular keratotic plugs, loss of follicular openings, perifollicular hyperkeratosis, telangiectatic vessels and red dots. (**c**) Horizontal section (4× magnification): dense peri-isthmus, peri-eccrine and perivascular lymphocytic infiltrate. (**d**) Higher magnification (10× magnification): vacuolar interface change at the dermoepithelial junction with folliculocentric inflammation. (**e**) Vertical section (4× magnification): perifollicular and perivascular lymphocytic inflammation extending into the deep dermis. (**f**) Vertical section (20× magnification): infundibular dilation with interface change and a dense perifollicular infiltrate. Panels (**c**–**f**) haematoxylin and eosin. Scale bars: (**c**) 1 mm, (**d**) 200 µm, (**e**) 1 mm, (**f**) 100 µm. These images are from the personal histopathological collection of Dr. Cosimo Misciali (Dermatology Unit, IRCCS Azienda Ospedaliero-Universitaria di Bologna, Italy) and illustrate the scalp biopsy findings of discoid lupus erythematosus described in the main text.

Among the lymphocytic subtypes, LPP shows lichenoid interface dermatitis at the infundibulum and isthmus, with hypergranulosis and Civatte bodies; FFA shares these features but has a different distribution and characteristically involves vellus follicles [40,53,54,55]. DLE shows patchy vacuolar interface dermatitis and a folliculocentric infiltrate that extends both superficially and deeply, often reaching the eccrine glands, together with basement membrane thickening and dermal mucin [22,56,57,58,59,60,61,62,63,64,65]. CCCA, the most common scarring alopecia in women of African descent, is defined by premature desquamation of the inner root sheath, concentric lamellar perifollicular fibrosis, and variable isthmus-level lymphocytic inflammation. One important caveat is that CCCA and LPP can be histopathologically indistinguishable, which makes the clinical context decisive (Figure 4 and Figure 5) [17,40,53,56,66].

Sebaceous gland atrophy (SGA) merits mention as a histopathologic finding whose diagnostic significance has been reassessed in recent years. It has been proposed as a potential contributor to hair loss in psoriasis, in which the sebaceous glands within the plaque undergo marked atrophy [67]. Rather than a marker of a single disease, it is better regarded as a reaction pattern and has been reported in early FFA, where it can precede overt pilosebaceous destruction [53,68]. On horizontal sections, atrophic sebaceous lobules appear as shrunken basaloid aggregates and may be mistaken for telogen germinal units; awareness of this pitfall reduces the risk of misclassification. When SGA is found in a biopsy that does not otherwise fit the psoriatic spectrum, the differential diagnosis should be broadened accordingly and the clinical history reviewed for relevant topical or systemic exposures [68].

Among neutrophilic cicatricial alopecia, FD is characterized by neutrophilic folliculitis and follicular rupture, with mixed inflammation and fibrosis developing in later lesions; DC typically shows deeper follicular rupture, suppurative inflammation, and abscess formation [8,10,19,20,69].

**Figure 6:**
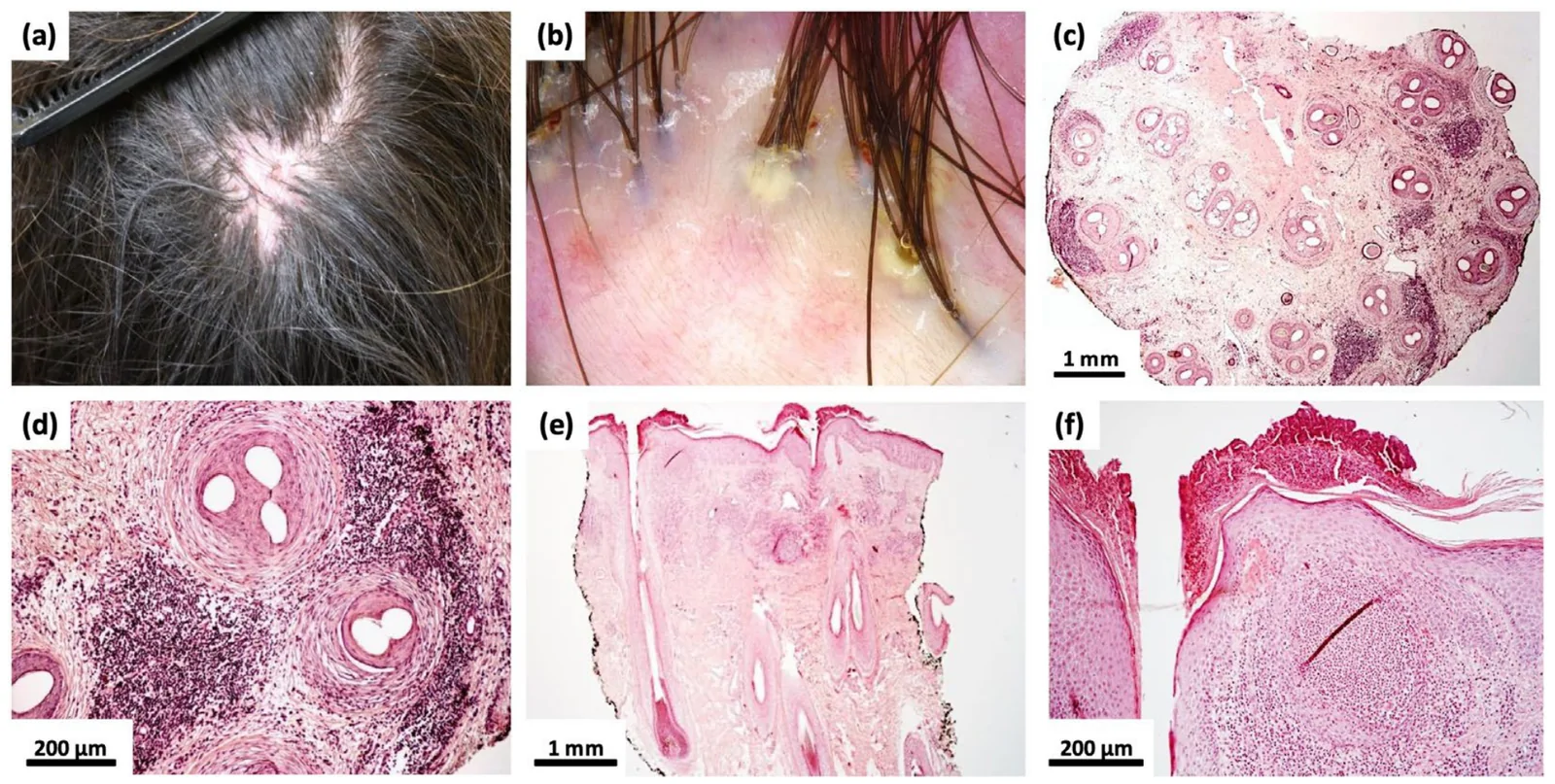
Folliculitis decalvans. (**a**) Clinical image: alopecic patch with tufted hairs. (**b**) Trichoscopy: perifollicular pustules, tufted hairs with more than six shafts emerging from a single follicular opening and radially arranged elongated vessels. (**c**) Horizontal section (4× magnification): follicular tufting with dermal fibrosis around isthmus-level follicles. (**d**) Higher magnification (10× magnification): dense, diffuse suppurative and mixed inflammatory infiltrate. (**e**) Vertical section (4× magnification): follicular rupture with suppurative inflammation reaching the epidermis. (**f**) Vertical section (10× magnification): tufted follicles surrounded by fibrosis and a mixed infiltrate. Panels (**c**–**f**) haematoxylin and eosin. Scale bars: (**c**) 1 mm, (**d**) 200 µm, (**e**) 1 mm (**f**) 200 µm. These images are from the personal histopathological collection of Dr. Cosimo Misciali (Dermatology Unit, IRCCS Azienda Ospedaliero-Universitaria di Bologna, Italy) and illustrate the scalp biopsy findings of folliculitis decalvans described in the main text.

Acne keloidalis nuchae is classified as a mixed cicatricial alopecia because both neutrophilic and lymphocytic inflammation may be present [10,18,19,20]. When the inflammatory pattern is ambiguous, clinical data are essential and may be decisive for the final diagnosis (Figure 6) [8].

Fibrosing alopecia in a pattern distribution (FAPD) is an underdiagnosed form of lymphocytic cicatricial alopecia, generally considered within the spectrum of LPP. First described around the turn of the millennium, FAPD combines the histopathologic features of LPP, namely vacuolar or lichenoid interface dermatitis at the infundibulum and isthmus, perifollicular fibrosis, and loss of sebaceous lobules, with the follicular miniaturization expected in AGA [54,55]. Because its clinical presentation is subtle and often asymptomatic, FAPD is easily misclassified as AGA with coincidental seborrheic dermatitis; trichoscopy, which shows anisotrichia, loss of follicular ostia, and perifollicular casts, provides the key prompt to biopsy [55]. Vacuolar or lichenoid interface change is the main histopathologic discriminator: FAPD, like other LPP subtypes, shows it, whereas CCCA does not, even when the two conditions share the same clinical picture of centroparietal hair loss with miniaturization [55,70,71]. In a large single-center series spanning ten years, FAPD accounted for 18% of scarring alopecia, a frequency well above the rate at which it is recognized clinically [72].

## 4. The Role of Scalp Biopsy: Indications, Sampling and Processing

### 4.1. Indications for Scalp Biopsy

Biopsy is warranted when alopecia remains diagnostically uncertain after clinical examination and trichoscopy, when a scarring process must be confirmed or excluded, or when objective documentation is required before starting systemic therapy [4]. Its principal indication is the evaluation of suspected primary cicatricial alopecia, in which histopathologic examination can confirm the diagnosis, classify the inflammatory subtype, and guide treatment [6,7].

In diagnostically challenging nonscarring alopecia, horizontal sections permit a quantitative assessment of follicular parameters that can aid the differentiation among chronic TE, FPHL, AGA and AA [21,24,73].

In AA, biopsy shows the characteristic peribulbar lymphocytic infiltrate (the “swarm of bees” appearance) together with increased catagen and telogen counts [43,51].

AAI is a condition in which clinicopathologic and trichoscopic correlation is essential. It produces acute diffuse shedding without the classic patches; its trichoscopic hallmarks are diffuse yellow dots and short regrown hairs [74]. The histopathologic changes, often subtle peribulbar infiltrates, can be interpreted correctly only when the pathologist is guided by trichoscopic suspicion [30,31]. These horizontal-section findings, reported in a series of 46 cases, are described in Section 3.1 [43]. A five-year study of AAI and diffuse AA likewise found trichoscopy to be indispensable for directing the biopsy to the most informative area and for distinguishing these two entities [75]. These observations indicate that clinical experience, but especially the trichoscopic ability and connection to the dermatopathologist, are essential.

Biopsy is also informative in suspected trichotillomania when the clinical history is unclear. Histopathologic clues include increased catagen follicles, pigment casts, traumatized hair bulbs, and trichomalacia [47,48]. The same quantitative approach applies to diagnostically challenging diffuse nonscarring alopecia [21,22,24,27,28,76].

Scalp biopsy has also become relevant in alopecia associated with immune checkpoint inhibitors, which can cause both nonscarring and scarring hair loss. In a systematic review of alopecia associated with these agents, AA was the most frequently reported pattern, and hair loss generally developed within the first year of treatment [77]. Drug-induced diffuse AA has likewise been reported with other agents, including the anti-CD30 antibody-drug conjugate brentuximab vedotin, in which trichoscopic and histopathologic confirmation was critical to perform the correct diagnosis [78].

Biopsy should be considered whenever the result is likely to resolve diagnostic uncertainty or influence treatment decisions. Careful patient selection is therefore central to maximizing the diagnostic yield of the procedure.

### 4.2. Selection of the Biopsy Site

Appropriate site selection is essential for diagnostic accuracy. Even a technically adequate biopsy may be uninformative if it is taken from an area of advanced follicular destruction, where only nonspecific fibrosis and follicular dropout remain (Table 1) [4,6]. In scarring alopecia, completely bald areas should be avoided, as they usually show end-stage changes of little diagnostic value [4].

Trichoscopy has markedly improved site selection. In cicatricial alopecia, findings such as perifollicular concentric white scales, follicular tufting, peripilar grey-white halos, follicular red dots, and keratotic plugs can guide sampling toward the most informative area [30,32]. Pruritus, burning, or a positive hair pull test offer additional guidance.

In scarring alopecia, the optimal site is the margin of the alopecic patch, the transition zone between affected and relatively preserved scalp, where inflammatory activity is greatest [4,7]. A well-chosen specimen should contain both scarred follicular units and follicles still undergoing active inflammation, since this combination maximizes diagnostic information [4,7]. In LPP and FFA, areas with perifollicular erythema or scaling should be targeted; in FD, zones with tufted hairs, perifollicular scaling, or follicular pustules; and in CCCA, the active peripheral edge of the vertex patch with peripilar white or grey halo [8,30,56,57,58,69,79].

For AA, an area showing trichoscopic evidence of active disease should be selected; histopathologic inflammation may be sparse or absent in chronic lesions, in which follicular miniaturization and hair-cycle changes may predominate [39]. The vertex or mid-scalp is preferred for diffuse alopecia such as AGA, chronic TE and FPHL [21]. Whatever the diagnosis, the biopsy site should be documented photographically, and the image should be sent to the dermatopathologist [4].

The rationale underlying these recommendations is common to all diagnoses. Trichoscopic signs such as perifollicular erythema, perifollicular concentric white scaling or casts, follicular tufting, peripilar grey-white halos, follicular red dots, keratotic plugs, black dots and broken hairs identify follicular units in which the disease process is still active and in which the follicle has not yet been replaced by fibrous tissue. Sampling these areas therefore maximizes the probability that the specimen contains the inflammatory and structural changes on which the diagnosis rests, whereas a completely bald or trichoscopically featureless area is likely to yield only nonspecific end-stage fibrosis, a finding compatible with any cicatricial alopecia and diagnostic of none. Table 1 indicates, for each condition, the trichoscopic marker that identifies this active zone [4,6,7,30,32].

### 4.3. Biopsy Technique

The 4-mm punch is the standard instrument. Its cross-sectional area (~13 mm^2^) is nearly twice that of the 3-mm punch (~7 mm^2^), and this difference translates directly into the number of follicular units available for reliable quantitative analysis [4,5,59]. The punch should be oriented parallel to the direction of hair growth to minimize follicular transection, and it must reach the subcutaneous fat; otherwise the anagen bulbs are amputated rather than sampled (Table 2) [59].

The St John’s protocol uses two 4-mm punch biopsies. In nonscarring alopecia, one biopsy is obtained from the clinically affected scalp, usually the vertex, and the other from an uninvolved control site; both are processed horizontally [80]. In cicatricial alopecia, both biopsies are obtained from the affected area: one is sectioned horizontally, whereas the other is bisected vertically, with one half processed in hematoxylin–eosin and the other reserved for DIF when indicated [80]. When trichoscopy-guided biopsy is routine, however, a single well-targeted specimen usually suffices.

In the two-biopsy approach, vertical sections are useful for assessing interface changes and deeper inflammatory processes, whereas horizontal sections facilitate the detection of focal follicular involvement and the evaluation of follicular abnormalities in scalp lupus [60,61]. Horizontal sections also permit the simultaneous assessment of multiple follicles and quantitative analysis of follicular parameters [27].

**Figure 7:**
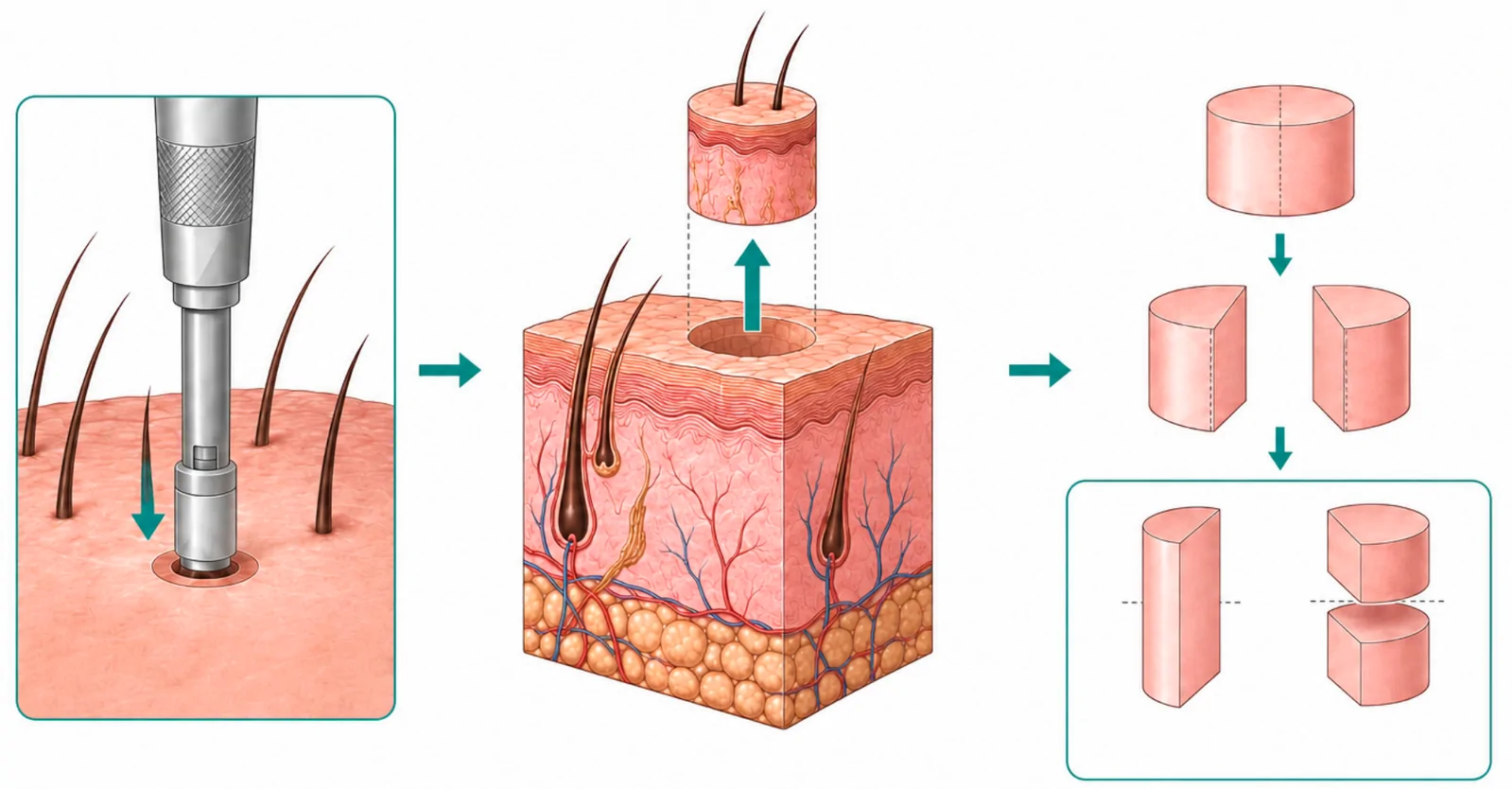
Tyler punch scalp biopsy technique: After local anesthesia, a 4-mm punch biopsy is used to obtain a cylindrical core of scalp tissue, oriented parallel to the direction of hair growth and extending into the subcutaneous fat. The specimen is then bisected longitudinally, and one half is subsequently sectioned transversely, generating corresponding tissue fragments that enable both horizontal and vertical histopathologic evaluation from a single biopsy specimen (Tyler technique). Figure 7 was created using a generative artificial intelligence tool (ChatGPT, version 5.6; OpenAI, San Francisco, CA, USA).

Available evidence generally supports combined approaches. One pilot study reported 100% sensitivity for transverse sections in nonscarring alopecia, whereas vertical sections performed better for LPP [62]. A meta-analysis found no significant overall difference in diagnostic yield between the two planes, whereas a retrospective series of 228 biopsies concluded that their combination maximized diagnostic yield [22,29]. Techniques that obtain both planes from a single specimen, such as the horizontal-vertical combined sectioning (HoVert) technique and the Tyler technique, offer the most efficient approach (Figure 7). The two methods differ in the way the specimen is divided. In the HoVert technique, the punch is first transected horizontally about 1 mm below the skin surface: the epidermal disc is bisected and embedded to provide vertical sections of the epidermis and of the dermoepidermal junction, while the deeper portion is serially sectioned horizontally, so that the full width of the biopsy remains available for follicular counts [65]. In the Tyler technique, the specimen is bisected vertically along its long axis and one half is then sectioned transversely, which is technically simpler but leaves only half of the punch for quantitative follicular assessment [63]. In our experience, the Tyler technique is practical and yields both horizontal and vertical information from a single specimen without compromising histologic quality, and its simplicity makes it well suited to routine use [63].

Local anesthesia is achieved with lidocaine, with or without epinephrine (2–3 mL), injected into the dermis and superficial fat. After injection, several minutes should elapse before the incision is performed, allowing vasoconstriction to provide adequate hemostasis. Infiltration directly into the biopsy site should be kept to a minimum, because distortion of follicular architecture is a real and underappreciated source of interpretive error [4]. The wound is closed with simple interrupted sutures, which are removed at approximately 10 days.

### 4.4. Specimen Processing and Histopathologic Evaluation

**Figure 8:**
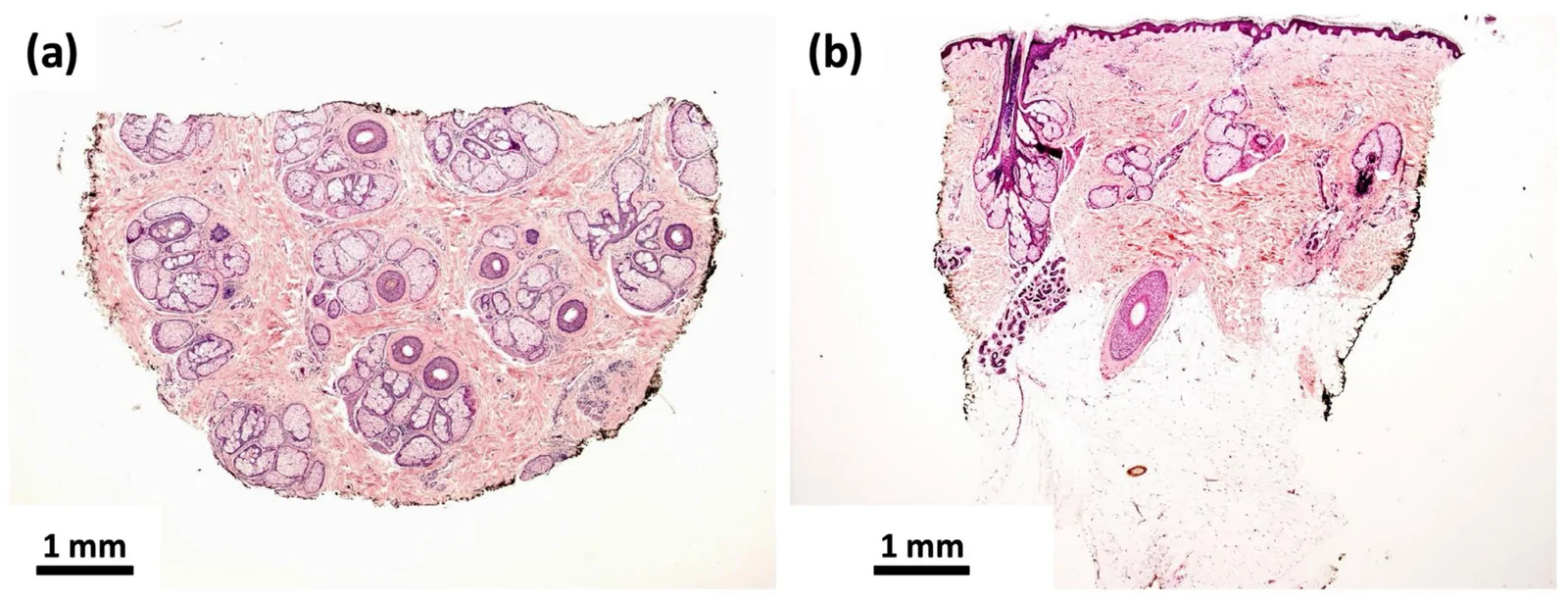
(**a**) Horizontal (transverse) section (4× magnification): multiple follicular units are visible in one plane, allowing follicular counts and the terminal-to-vellus ratio to be determined. (**b**) Vertical (longitudinal) section of the same specimen (4× magnification): the full profile from epidermis to subcutis is preserved, but only a few follicles are represented. Both panels are reproduced at the same scale. Haematoxylin and eosin. Scale bars: (**a**,**b**) 1 mm. These images are from the personal histopathological collection of Dr. Cosimo Misciali (Dermatology Unit, IRCCS Azienda Ospedaliero-Universitaria di Bologna, Italy) and illustrate the difference between horizontal and vertical sectioning of a scalp punch biopsy described in the main text.

Orientation of the specimen before sectioning is as important as the excision itself. Horizontal sections are obtained by cutting parallel to the skin surface and permit the simultaneous evaluation of multiple follicles [48]. This approach quantifies the parameters needed to differentiate nonscarring alopecia: follicular density, terminal-to-vellus hair ratio, hair-cycle distribution and the degree of perifollicular fibrosis (Figure 8) [21,22,27,28,64].

In normal scalp, approximately 85–90% of follicles are in anagen, and telogen hairs account for less than 10–15%. On horizontal sections, a terminal-to-vellus ratio below 4:1 at the isthmus is generally regarded as supportive of AGA. An increased proportion of telogen follicles, in the setting of a preserved terminal-to-vellus ratio, supports TE [21,22,27,28,36]. Because these changes may be subtle, quantitative findings should be interpreted together with the clinical presentation.

Proper grossing is a prerequisite for reliable histopathologic evaluation. In one large series, 22 of 228 biopsies (9.6%) were inconclusive because of faulty grossing, which indicates the need for trained laboratory personnel [22].

A further consideration in quantitative histopathologic assessment is that follicular density is not uniform across the normal scalp. A cadaveric study of 240 specimens from four anatomic zones found that the temporoparietal area showed the lowest total hair and follicular-unit counts, with approximately 18 total hairs per 4-mm punch compared with approximately 24 at the vertex [81]. The anagen-to-telogen ratio was comparable across scalp regions, and no significant associations with sex or increasing age were identified [81]. These findings indicate that biopsy location should be considered when interpreting quantitative follicular parameters.

### 4.5. Ancillary Diagnostic Techniques

Hematoxylin-eosin staining remains the cornerstone. Ancillary techniques may be used when routine histopathologic examination does not resolve the diagnostic uncertainty [4].

Direct immunofluorescence (DIF) is most useful when lupus erythematosus is in the differential diagnosis. Granular deposits of immunoglobulins and complement along the basement membrane zone may support a diagnosis of DLE and help distinguish it from LPP, which can closely mimic both clinically and histopathologically [40,53,54,82].

Periodic acid-Schiff (PAS) staining identifies fungal organisms in hair shafts and follicular structures in suspected tinea capitis [4]. Elastic fiber stains (e.g., Verhoeff-Van Gieson) distinguish follicular streamers from true follicular scars on horizontal sections [6,83]. In FD, bacterial culture of active pustular lesions may identify *Staphylococcus aureus* or other organisms and guide antimicrobial selection, although culture findings are supportive rather than diagnostic [8,84,85].

Non-invasive methods are increasingly used alongside biopsy: quantitative trichoscopy provides non-invasive measurements of hair parameters; however, the values obtained are not directly interchangeable with histopathologic measurements [86].

For now, accurate biopsy interpretation still requires the integration of clinical, trichoscopic, and histopathologic information.

## 5. The Trichoscopic-Histopathologic Correlation as a Diagnostic Paradigm

Contemporary diagnostic practice in hair disorders rests on the integration of trichoscopy and histopathology, a combination that has changed the approach to uncertain alopecia. Trichoscopy is not merely a tool for choosing the biopsy site; it generates the diagnostic hypothesis that shapes how the pathologist reads the specimen. In conditions with subtle or overlapping histopathologic findings, the clinical context provided by the trichoscopist can determine whether the specimen is interpreted correctly or yields a nondiagnostic report.

Our experience from the University of Bologna, built over decades of combined trichoscopic and histopathologic assessment, illustrates this point. AAI, a condition that may not fulfill classical histopathologic criteria and can easily escape recognition, could be reliably recognized only with integration of clinical, trichoscopic, and histopathologic findings. The trichoscopist identified diffuse yellow dots, pigtail hairs, and short regrowing hairs and shared these findings with a pathologist who might otherwise see only nonspecific miniaturization [43,74,75]. Histopathologic evaluation provides the additional quantitative and morphologic criteria described in Section 3.1 [43].

A subsequent five-year study concluded that trichoscopy was essential both for separating AAI from diffuse AA and for targeting the most informative biopsy site [75]. More recent work found that reflectance confocal microscopy shows substantial agreement with histopathology in AAI; this suggests that non-invasive imaging may eventually complement, though not replace, the trichoscopic-histopathologic correlation [87].

An important corollary concerns FFA in particular. Trichoscopy detects perifollicular erythema and scaling during active disease, yet some patients show progressive scarring hair loss without any trichoscopic evidence of ongoing inflammation. A cross-sectional study of LPP and FFA showed that trichoscopic findings do not consistently reflect the degree of histopathologic inflammation: cases with dense perifollicular lymphocytic infiltrates on biopsy may appear trichoscopically quiescent [88]. Moreover, in FFA, progression of hairline recession may occur despite absent or improving inflammatory signs [89]. One proposed explanation is that the lymphoid infiltrate in FFA tends to lie deeper than in classic LPP, below the resolution of surface trichoscopy [90]. This has a clear clinical consequence: histopathology is needed to characterize and quantify inflammatory activity in FFA even when the trichoscopic picture is reassuring, and treatment decisions should not rely on trichoscopy alone in this setting.

## 6. Diagnostic Pitfalls

The most frequent pitfall is sampling end-stage follicular destruction, where only nonspecific fibrosis remains, and the underlying cause cannot be determined [4,5,6]. Equally problematic is insufficient biopsy depth: anagen follicular bulbs lie in the subcutis, and superficial specimens may miss both these structures and the deep peribulbar inflammatory infiltrates that characterize conditions such as AA [4,5,22,27,39,59].

Punches smaller than 4 mm reduce the number of follicular units available for quantitative assessment, and improper specimen orientation during processing produces oblique sections that distort follicular counts [4,5,22,59,60]. Several lymphocytic scarring alopecias share overlapping features; CCCA and LPP, for example, may be histologically and immunophenotypically indistinguishable, making clinicopathologic correlation essential [7,66,91]. The three-way differential of AGA, TE, and AA on biopsy alone remains notoriously difficult [24].

A related and underappreciated challenge is multifactorial alopecia, the coexistence of two or more diagnoses in a single biopsy specimen. In a large consecutive series of 1360 biopsies, more than one diagnosis was identified in 12.5% of specimens; the most frequent combination was AGA with CCCA, followed by CCCA with end-stage TA; three concurrent diagnoses, AGA with CCCA and end-stage TA, accounted for 3.5% of the multifactorial cases [92]. These figures call for two practical adaptations. First, biopsy-site selection should target a watershed zone, an area of the scalp where the disease processes under consideration are known to overlap anatomically; when CCCA, FPHL, and TA are being evaluated together, for instance, the frontotemporal aspect of the crown lies at the intersection of all three [68,92]. Second, horizontal sections are indispensable in this setting: because different follicular units within the same 4-mm punch may be affected by different processes, only transverse sectioning allows them to be assessed simultaneously and side by side [68,92].

The terminology used in the pathology report also warrants attention. “Consistent with” or “compatible with” signal high diagnostic confidence, whereas “suggestive of” or “cannot exclude” indicate residual uncertainty that may require further workup [4].

## 7. Practical Diagnostic Algorithm

**Figure 9:**
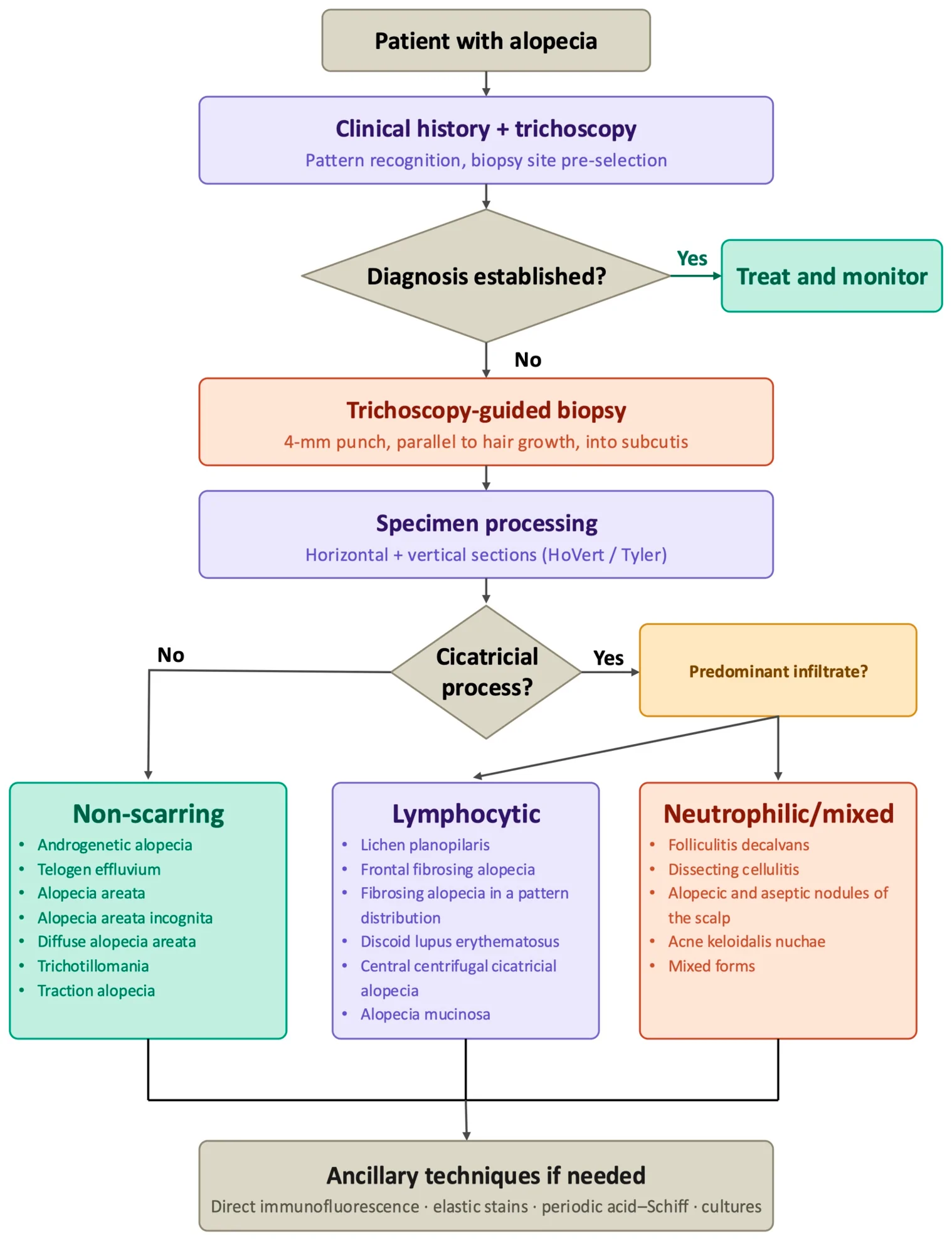
Stepwise diagnostic algorithm for scalp biopsy in alopecia. After clinical history and trichoscopic assessment, biopsy is indicated when the diagnosis remains uncertain, when a scarring process cannot be excluded, or when systemic treatment requires histopathologic documentation. A 4-mm punch biopsy, oriented parallel to the direction of the hair shaft and extending into the subcutaneous fat, is processed by combined horizontal and vertical sectioning (HoVert or Tyler technique). Histopathologic findings distinguish scarring from nonscarring forms; in scarring alopecia, the predominant inflammatory cell type guides subclassification according to the NAHRS criteria. Ancillary techniques, including direct immunofluorescence, elastic fiber stains, PAS staining, and microbiologic cultures, are performed selectively when standard hematoxylin-eosin evaluation is inconclusive.

The first question is whether the alopecia is scarring or nonscarring, a distinction of major prognostic and therapeutic importance (Figure 9). Loss of follicular ostia, absence of vellus hairs, and dermal sclerosis on examination, together with trichoscopic findings such as fibrotic white dots or absent follicular openings, point toward a scarring process.

When clinical examination and trichoscopy are inconclusive, scalp biopsy should be considered. The sectioning method should reflect the clinical suspicion: horizontal sections are particularly useful in nonscarring alopecia, whereas combined horizontal and vertical evaluation, when feasible, maximizes the available diagnostic information [22,29,62]. Ancillary techniques (DIF, PAS, elastic stains, cultures) are added when standard evaluation leaves the diagnosis open. Throughout, close collaboration between the dermatologist and the dermatopathologist is essential for accurate diagnostic interpretation; this collaboration is what ultimately turns a tissue sample into a diagnosis [4].

## 8. Conclusions

Scalp biopsy remains decisive in diagnostically uncertain alopecia, and its yield depends on appropriate patient selection, trichoscopy-guided site identification, adequate technique, and careful specimen processing. Combined horizontal and vertical sectioning obtained from a single specimen, together with selectively applied ancillary studies, allows the most complete assessment of follicular architecture and inflammatory patterns. Sustained collaboration between the dermatologist and the dermatopathologist is what ultimately turns a tissue sample into a diagnosis.

## Figures and Tables

**Table 1:** Recommended biopsy site according to alopecia type. In each entry, the indicated site is the area in which trichoscopy identifies ongoing follicular inflammation or early follicular damage, that is, the region most likely to contain diagnostic changes rather than end-stage fibrosis.

Alopecia Type	Recommended Biopsy Site
Lichen planopilaris	Trichoscopy-guided area showing perifollicular concentric white scaling or casts, preferably within an active margin.
Frontal fibrosing alopecia	Trichoscopy-guided area of the frontal hairline showing perifollicular concentric white scaling or casts.
Discoid lupus erythematosus	Active trichoscopically abnormal area showing follicular red dots, keratotic plugs, or perifollicular concentric white scaling.
Folliculitis decalvans	Trichoscopy-guided area showing follicular tufting, preferably near an active pustular or inflammatory area.
Central centrifugal cicatricial alopecia	Trichoscopy-guided area showing a peripilar grey-white halo within or beyond the clinically apparent central involvement.
Alopecia areata	Area showing trichoscopic evidence of active disease, such as black dots, broken hairs, tapering or exclamation-mark hairs
Androgenetic alopecia/female pattern hair loss	Vertex or mid-scalp
Telogen effluvium	Vertex or mid-scalp
Alopecia areata incognita	Trichoscopy-guided area showing diffuse yellow dots, short regrowing hairs, or pigtail hairs.
Diffuse alopecia areata	Trichoscopy-guided area showing dystrophic hairs, black dots, broken hairs, or tapering hairs.
Fibrosing alopecia in a pattern distribution	Trichoscopy-guided area within the pattern alopecia showing perifollicular erythema, casts or scaling, loss of follicular openings, and miniaturization.
Traction alopecia	Area showing established hair loss at the clinically involved margin; in suspected end-stage disease, sample an area with residual follicles rather than completely smooth scarred scalp.
Trichotillomania	Clinically affected area showing hairs of different lengths, broken hairs, or other signs of recent traumatic activity.
Dissecting cellulitis of the scalp	Active inflammatory area at the margin of a nodule, abscess, or boggy plaque, avoiding a completely suppurated or end-stage scarred center.
Acne keloidalis nuchae	Active papular or pustular margin containing residual follicles, rather than the center of a mature keloidal plaque.
Multifactorial alopecia	Trichoscopy-guided watershed area in which the suspected processes overlap; for CCCA, FPHL, and traction alopecia, the frontotemporal aspect of the crown has been proposed

**Table 2:** Technical recommendations for scalp biopsy in alopecia.

Parameter	Recommendation
Punch size	4 mm
Depth	Into subcutaneous fat
Number of biopsies	One with Tyler technique
Orientation	Parallel to hair growth
Sectioning	Combined horizontal + vertical; with Tyler technique
Anesthesia	Lidocaine ± epinephrine
Closure	Simple interrupted sutures (removal at 10 days)

## Data Availability

No new data were created or analyzed in this study. Data sharing is not applicable to this article. Figure 1, Figure 2, Figure 3, Figure 4, Figure 5 and Figure 6 are from the personal histopathological collection of Dr. Cosimo Misciali (Dermatology Unit, IRCCS Azienda Ospedaliero-Universitaria di Bologna, Italy), and these figures are only intended to illustrate existing conclusions, rather than to support the inference of new conclusions.

## Abbreviations

The following abbreviations are used in this manuscript: AA: alopecia areata; AGA: androgenetic alopecia; AAI: alopecia areata incognita; CCCA: central centrifugal cicatricial alopecia; DC: dissecting cellulitis; DIF: direct immunofluorescence; DLE: discoid lupus erythematosus; FAPD: fibrosing alopecia in a pattern distribution; FD: folliculitis decalvans; FFA: frontal fibrosing alopecia; FPHL: female pattern hair loss; TA: Traction Alopecia; TE: telogen effluvium; HoVert: horizontal-vertical combined sectioning technique; LPP: lichen planopilaris; NAHRS: North American Hair Research Society; PAS: periodic acid-Schiff; PFU: pauci-follicular unit; SGA: sebaceous gland atrophy.
